# Utility of PHH3 in Evaluation of Mitotic Index in Breast Carcinoma and Impact on Tumor Grade

**DOI:** 10.31557/APJCP.2020.21.1.63

**Published:** 2020

**Authors:** Elham Mirzaiian, Zahra Sadat Tabatabaei Ghods, Seyed Mohammad Tavangar, Binesh Emami, Maryam Oraei, Roya Safyari, Hiva Saffar

**Affiliations:** 1 *Department of Pathology, Shariati Hospital, Tehran University of Medical Sciences, *; 2 *Anatomical and Clinical Pathologist, Farvardin Laboratory, *; 3 *Private Center, Tehran, Iran. *

**Keywords:** Breast carcinoma, mitosis, Phosphohistone H3

## Abstract

**Background:**

Mitotic activity index is considered as the most important grading component to predict prognosis in invasive breast carcinoma. But it is believed that it is also the cause of discordance in grade estimation based on Bloom-Richardson system. Thus, reproducible methods such as immunohistochemistry (IHC) based analysis methods appears to be of great value in facilitating mitotic count.

**Materials and Methods:**

In the present study, we examined the utility of Phosphohistone H3 by IHC in various grades of breast carcinoma and compared it with traditional mitotic count by hematoxylin and eosin (H and E) staining and probable changes in tumor grading.

**Results:**

Total 90 cases of invasive breast carcinoma were evaluated. Mean mitotic count were 8.6 and 6.4/10HPF in IHC and HandE groups, respectively. Although , mean average count was higher by IHC method , good correlation was observed(R=0.914). Using PHH3 IHC, two out of 33 cases of grade I tumors were upgraded in to grade II and three cases of grade II were upgraded in to grade III. None of the tumors were down graded.

**Conclusion:**

Similar to some other previous studies, we found PHH3 a robust sensitive and practical marker for mitotic count in breast carcinoma. Especially it is helpful to identify the most proliferating area. However, further studies are required to confirm the superiority of this biomarker for including in grading system.

## Introduction

The intrinsic biological characteristics of invasive breast carcinoma are related to histologic grade (Cui et al., 2015). Mitotic activity index is included in grading system and considered as the most important component to predict prognosis (Kim et al., 2017). It is believed that mitotic count difference is the most common cause of discordance in grade estimation based on Bloom-Richardson system (Woo et al., 2015).The low reproducibility in mitotic count could be due to difficulty in identification of mitotically active areas in HandE staining or mitotic mimickers such as hyperchromatic nuclei, karyorrhectic or apoptotic cells (Kim et al., 2017). Whereas cells in prophase usually are not counted in routine hematoxylin and eosin (Cui et al., 2015). Moreover, measuring the Mototic Activity Index (MAI) is time consuming (Lee et al., 2014) and based on the number of mitosis per unit area, so inherently confounded by tumor cellularity (Gerring et al., 2015). Thus, reproducible methods such as immunohistochemistry based analysis methods appears to be of great value in facilitating mitotic count in breast carcinoma grading and subsequent treatment decision (Cui et al., 2015; Sillem et al., 2017).

Ki67 is a DNA binding nuclear protein expressed in all active phases of cell cycle (G1,S, G2 but not G0), which is widely used and been accepted as a reliable quantitave indcator for proliferation (Cui et al., 2015; Kim et al., 2017). However, there are some doubts in utility of Ki67 as being representative of proliferation index. Because cells in G1 phase have shown uncertain destinies (Williams and Stoeber, 2012; Kim et al., 2017).

Histone H3 is one of the five histone proteins which together form the major proteins constituents of chromatin in eukaryotic cells.The mitosis marker anti-phosphohistone H3 was first introduced in 1997 (Hendzel et al., 1997; Nakashima et al., 2013). Antibodies directed against phosphorylated histone H3 reveals that modification is almost exclusively expressed in actively proliferating cells during M phase (Gerring et al., 2015 ) and is not observed during apoptosis (Sillem et al., 2017).

Utility of PHH3 as mitosis indicator has been evaluated in various tumors including melanoma (Casper et al., 2010; Ikenberg et al., 2012; Ladstein et al., 2012; Tetzlaff et al., 2013; Cui et al., 2015), neuroendocrine tumor (Tsuta et al., 2011; Cui et al., 2015), colorectal adenocarcinoma, ovarian serous carcinoma, smooth muscle tumors, astrocytoma and meningioma (Ribalta et al., 2004; Colman et al., 2006; Nasr and El-Zammar, 2008; Casper et al., 2010, Tsuta et al., 2011; Tetzlaff et al., 2013; Kim et al. 2017), and revealed correlation with outcome (Ribalta et al., 2004; Colman et al.,2006; Nasr and El-Zammar, 2008; Casper et al., 2010; Tsuta et al., 2011; Tetzlaff et al., 2013; Kim et al., 2017).

In a study conducted by Cui et al., (2015), MAI was strongly corelated with PHH3 and they proposed that PHH3 could potentially be helpful in breast cancer grading. 

In the present study, we examined utility of PHH3 in various grades of breast cancer and compared it with traditional mitotic count. Moreover, we evaluated any possible correlation between PHH3 and other histologic prognostic factors including hormone receptors and tumor size.

## Materials and Methods

**Figure 1:**
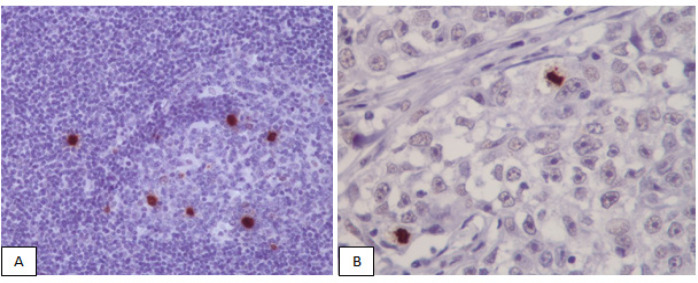
Mitotic Figures Highlighted by PHH3 in IHC.(A), Tonsil as positive control; (B), Breast ductal carcinoma

In this study 90 samples diagnosed as invasive breast carcinoma during 2015 to 2017 were evaluated. The slides and paraffin blockes were retrived from archive of pathology department of Shariati hospital, Tehran university of medical science. Slides with adequate tumoral tissue, without or with minimal necrosis or hemorrhage were included. Representative H and E slides were examined and graded according to Nottingham modification of the Scarff-Bloom-Richardson (MBR) scoring system.Mitotic figures were counted manually on HandE stained slide at High Power Field in 10 successive fields in the most mitotically active areas. Mitotic figures were defined as cells in prophase,metaphase or anaphases. Apoptotic cells were excluded. Then mitotic scores were assigned based on MBR criteria according to 0.55 mm microscope field diameter by H and E slide (Woo et al., 2015). PHH3 IHC results also were evaluated by the same method on the same day or the next day. Only PHH3 positive nuclei in the prophase,metaphase,anaphase and telophase were included. Finely granular staining of intact nuclear membrane was regarded as cells being in interphase and excluded (Cui et al., 2015; Sillem et al., 2017) (Figure-1). Moreover, data regarding hormonal profile, probable lymph node involvement and size of tumors were extracted. All data were analyzed using SPSS 22.0 soft ware.

## Results

Total 90 cases of invasive breast cancer were evaluated as follow Invasive ductal carcinoma:79 (87.8%); invasive lobular carcinoma:4 (4.4%); metaplastic carcinoma:3 (3.4%), micropapillarycarcinoma:2 (2.2%); papillary carcinoma:2 (2.2%). The mean avarage age of the patients was 54±12.5 years. Among them, 68 and 70 cases revealed mitosis by H and E method and IHC, respectively. Mean avarage (the lowest and highest range) of mitotic counts were 6.4 (0-26) and 8.6 (0-30) per10HPF in two groups, respectively. Although mean avarage was higher in IHC group, good correlation was observed between two methods (R=0.914).

Frequency of various grades of tumor, status of hormone receptors and HER2-neu expression and correlation with PHH3 are summerized in Table 1.

There was no significant correlation between positive Estrogen (ER) and Progestrone (PR) receptor expression and increase in mitotic count either by H and E or PHH3 counting method.

However, Significant correlation was observed between lymph node involvement or tumor size and mitotic count by PHH3 (p =0.01 for both parameters). Meaning that the mitotic count ( by PHH3 method), was higher in tumors with greater size or tumors with involvement of lymph nodes.

Using PHH3 IHC, two out of 33 cases of grade I tumors were upgraded in to grade II and three cases of grade II were upgraded in to grade III, (total 5.55% upgrade rate). None of the tumors were down graded.

**Table 1:** Average Numbe of Mitosis Counted in HandE Method and by PHH3 in Various Grades of Breast Cancer

Histologic grade	Number of Invasive cancers	Avarage count of mitosis/10HPF by PHH3	Avarage count of mitosis/10HPF in HandE method	Estrogen Receptor expression		Progestrone receptor expression		HER2-neu expression		
				Positive	Negative	Positive	Negative	Positive	Equivocal	Negative
I	33	5.21	4.78	32	1	29	4	33	0	0
II	33	7.85	5.71	28	5	21	12	31	1*	1
III	24	14.33	9.46	11	13	11	13	18	2*	4

*Based on IHC results, five tumors were classified as "Equivalent". However, further CISH study results were available for two of them, both negative for amplification.Thus, finally, three cases were categorized as “Equivocal”.

## Discussion

The proportion of proliferating tumor cells is an important variable in diagnostic surgical pathology (Lee et al., 2014). It represents tumor aggressiveness and play important role to guide treatment in some tumors including breast carcinoma (Lee et al., 2014). Mitotic index is a key component in histologic grading (Cui et al., 2015). Schwartz et al., (2014) evaluated a large series of breast cancer cases for the SEER program, and reported that histologic grade is the most important prognostic factor for overall survival, which magnify the significance of accurate histologic grading for breast carcinoma (Woo et al., 2015).

There are many methods of quantifying the proportion of proliferating cells (Lee et al., 2014). The traditional mitotic count is still widely used (Veta et al., 2015). The cells in M-phase can be identified by their characteristic morphology (Veta et al., 2015). However, it is time consuming and is not reproducible due to confounding factors even amongst trained pathologists (Lee et al., 2014).

Ki67 has been used as surrogate marker of proliferation and moreover, it acts as a prognostic factor, since increased Ki67 index has been correlated with shorter cancer survival (Woo et al., 2015). Although it is not specific for mitosis and is positive in G1,S,G2 and M phases (Woo et al., 2015). So, some other more specific IHC markers have been introduced, including PHH3 (Woo et al., 2015).

PHH3 has been shown as a useful indicator of mitosis and helpful marker in determining hot spots for mitotic activity evaluation (Woo et al., 2015; Kim et al., 2017). PHH3 targets cells in mitosis and theorically could match with mitotic count determined on HandE method.In practice, however, mitosis counted by PHH3 sometimes do not match with the HandE method (Kim et al., 2017).This fact partially could be explained by subjectivity associated with mitotic count or inaccurate localization of the most active areas (Kim et al., 2017). So, based on our findigs, not surprisingly, mean mitosis count by PHH3 method was higher in comparison to HandE method, which is in concordance with findings reported by (Cui et al., 2015). In our study, two cases were up graded from grade I to II and three case was upgraded from II to III. There was no change from I to III or down grading .In a study by Kim et al (Kim et al., 2017), 29 (13.2%) and 17 (7.2%) of cases were up and down graded, respectively. Because downregulation mainly was reported in older blocks, the authors thought that probably it could be due to loss of antigen preservation (Kim et al., 2017).In another study by Cui et al., (2015) 27%,41% and 33% of breast cancer cases were up graded by PHH3 based mitotic counting by 3 observers. Woo et al., (2015), reported 24% change in grading (107/451)including 101 up grading and 6 downgrading. Seven cases moved from grade I to grade III. Based on our findings, none of the cases moved from I to III. 

Significant correlation was observed between lymph node involvement or tumor size and mitotic count by PHH3. However, there was no correlation between ER or PR expression and PHH3. The findings are rather discordant with findings reported by Sillem et al (Sillem et al., 2017). They counted PHH3 immunostained mitosis /10HPF in breast cancer tissue from 72 patients before Neoadjuvant Chemotherapy. Based on their results, no significant correlation was found between size or nodal status before therapy and PHH3 expression. Invasive ductal carcinomas showed more PHH3 positive cells than invasive lobular carcinomas. Moreover, triple negative breast cancers showed>10 positive PHH3 mitotic cells significantly more frequent than luminal type breast cancer (p=0.003).

One important limitation of our study is lack of clinical outcome. Moreover, the data regarding clinical stage of the patients were not available.Based on study by Kim et al., (2017), PHH3 showed correlation with disease free survival (p=0.043) while Ki67 did not (p=0.356). Although the duration of follow up was not long (median : 46 months).

In conclusion, similar to other previous studies, we found PHH3 a robust sensitive and practical marker for mitotic count in breast carcinoma. Especially, it is helpful to identify the most proliferating area. However, further studies are required to confirm the superiority of this biomarker for including in grading systems.
